# *Neoscytalidium novaehollandiae* causes dieback on *Pinus eldarica* and its potential for infection of urban forest trees

**DOI:** 10.1038/s41598-022-13414-8

**Published:** 2022-06-04

**Authors:** Mehrdad Alizadeh, Naser Safaie, Masoud Shams-Bakhsh, Mohammad Mehrabadi

**Affiliations:** 1 Department of Plant Pathology, Faculty of Agriculture, Tarbiat Modares University, Tehran, Iran; 2 Department of Entomology, Faculty of Agriculture, Tarbiat Modares University, Tehran, Iran

**Keywords:** Microbiology, Plant sciences

## Abstract

*Neoscytalidium novaehollandiae* is one of the most important pathogens on woody plants which has increasingly been reported as a pathogen in different hosts in recent years. The pine trees are widely cultured in many cities of Iran. In recent years, dieback symptoms were observed on *Pinus eldarica* trees in Tehran and Qazvin provinces. The aim of this study was to investigate the dieback causal agent on *P. eldarica* trees in Iran. The branches and cones of *P. eldarica* trees were sampled for fungal isolation. The morphological and molecular characterizations (ITS, LSU, and TEF1-α regions) identified *N. novaehollandiae* as a dieback causal agent. This is the first report *of N. novaehollandiae* disease of *P. eldarica* trees in Iran. Furthermore, disease severity was assayed on 19 urban forest trees under three different temperature and relative humidity (RHs) regimes. C regime (29 °C and 15% RH) displayed more disease severity on detached branches than B (24 °C and 80% RH) and A (19 °C and 35% RH) ones. This study presents the host range of this pathogen, and showed that these potential hosts are prone to this pathogen under high temperature and low humidity which urban forest trees experienced in recent decades.

## Introduction

The genus “*Neoscytalidium”* is a member of Botryosphaeriaceae family and containing four species including *N. dimidiatum*, *N. oculus*, *N. orchidacearum*, and *N. novaehollandiae*^1^. This genus may potentially infect plants^2–4^, humans, and animals^5–7^. The distribution of this genus has extended to all continents. *Neoscytalidium* genus can influence the different parts of plants by causing diseases that show different symptoms on aerial and underground parts of hosts including dieback, elongated canker^8^, brown spot^9^, collar and root rot^10^, fruit internal brown rot^11^, stem and fruit canker^12^, leaf blight^13^, canker, shoot blight and fruit rot^14^, root rot^15^, black canker and root rot^16^, defoliation, root rot, inner stem necrosis, and plant death^2^, shoot and needle blight^17^, tuber rot^18^, black and dry root rot, and stem rot^19^, shoot blight^20^.

Among urban forest trees, coniferous trees are important in and around the cities. These trees are prone to decline since the conifer plantations have situations without seeds for the regrowth of trees^21^. *Pinus eldarica*, also known as the Tehran pine, is an evergreen coniferous tree that is native to Western Asia (adapted to warm and dry climates), involving Asia Minor, the Middle East, and land surrounding the Caspian Sea. *P. eldarica* grows in different types of soils, and different types of disease may occur on it^22^. This species has been introduced to Iran several hundred years ago, and it tolerates air contamination, dust, drought and cold^23^. In addition to *Coleosporium tussilaginis* which can infect *Pinus eldarica* with needle rust symptoms^24^, *Pinus eldarica* trees have several pathogens such as *Diplodia sapinea*^25^, *Phytophthora nicotiana* var. *parasitica*, *Pythium ultimum*, *Pythium paroecandrum*, *Rhizoctonia solani, Fusarium proliferatum*^26^, *Fusarium solani*^27^and *Microsphaeropsis olivacea*, *M. protea* and *Kalmusia variispora*^28^.

The temperature and humidity play essential roles in fungal behaviors in terms of pathogenicity and growth^29,30^. Disease severity is a significant parameter to calculate disease level representing infection degree, colonization and, damaged tissue^31^. The disease severity index refers to the effect of disease on the plant or its small parts. It can be utilized for the generalization of regional disease severity to a community, country, state, or nation. This index may likewise be applied for resistance to pathogens^32^. The pathogenicity tests in fungi, using detached branches inoculation, can be used as a tool for determining the potential host range of phytopathogenic fungi, and their acquired results may extend to determine foliar susceptibility, and quarantine and management recommendations against these potential pathogens in plants^33^.

Owing to the growing concern for pine trees in Tehran and Qazvin provinces as the disease incidence has increased in recent years ago, study was conducted to identify the causal agent of dieback disease on *P. eldarica* trees, and to evaluate its pathogenicity potential and virulence on detached branches of urban non-host forest trees in three different conditions in regard to the temperature and relative humidity.

## Results

### Isolates

The disease symptoms on the pine trees included green needle death, branch dieback, and decline. Approximately 80% trees in each region were infected. Twelve fungal isolates were recovered from 15 symptomatic samples collected from three regions (one arboricultural area in Qazvin and two ones in Tehran).

### Morphological features

**Figure 1:**
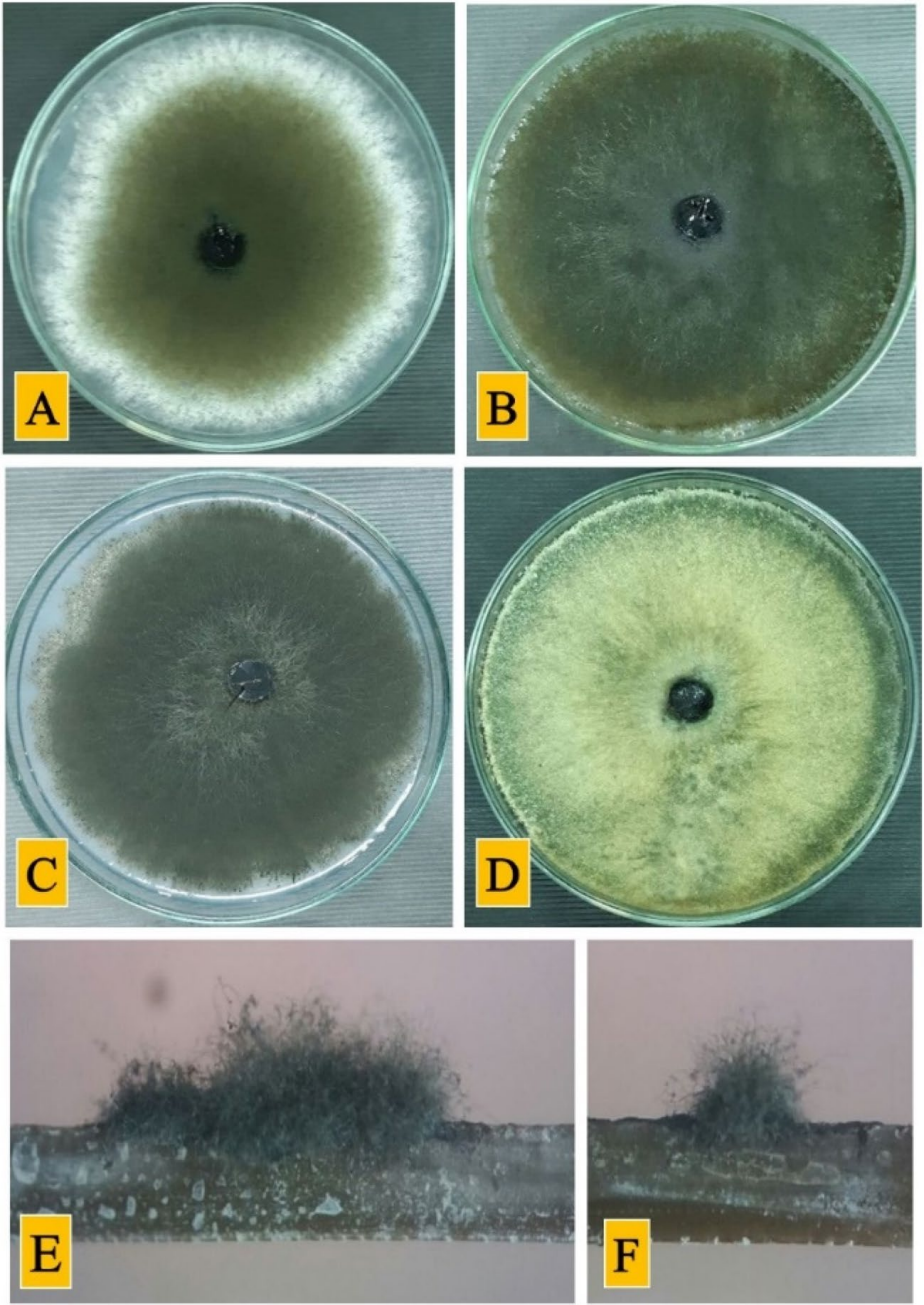
Fungi on potato dextrose agar (PDA) medium after 5 days (**A**); oatmeal agar (OA) medium after 8 days (**B**); potato carrot agar (PCA) medium after 8 days (**C**); malt extract agar (MEA) medium after 8 days (**D**); and conidiomata on pine needles in culture after 15 days (**E** and **F**.).

**Figure 2:**
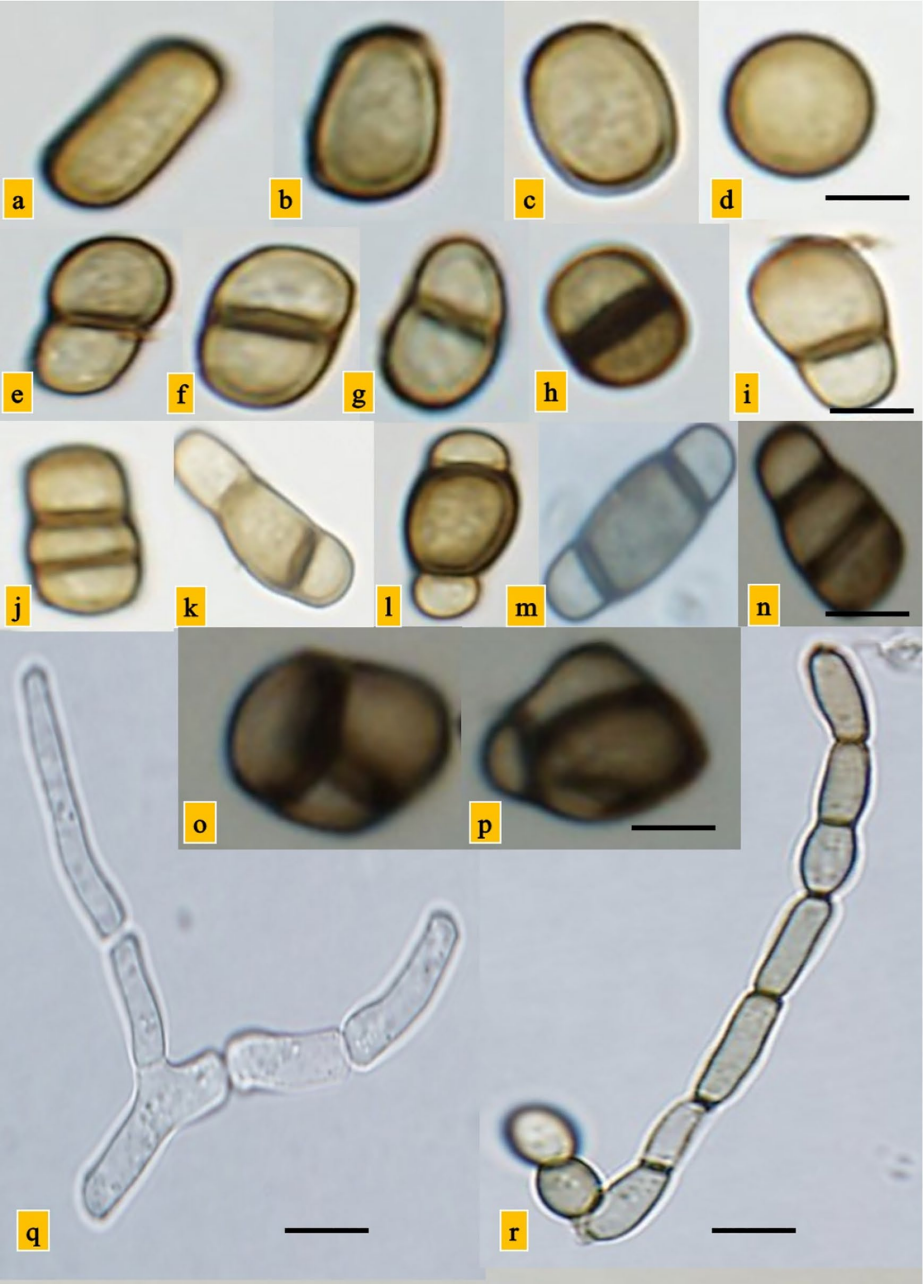
Spores of *Neoscytalidium novaehollandiae*; light brown aseptate conidia (**a**, **b**, **c** and **d**); light brown 1-septate conidia (**e**, **f**, **g**, **h** and **i**) light brown two-septate conidia (**j**, **k**, **l**, **m**, and **n**); muriform conidia (**o** and **p**); Chains of arthroconidia (**q** and **r**; q: premature of arthroconidia and r: mature of arthroconidia). Scale bars: a, b, c and d = 3 μm; e, f, g, h, i, j, k, l, m, and n = 4 μm; o and p = 2.5 μm; q and p = 5 μm.

Cultural characteristics of *N. novaehollandiae* grown on PDA, OA, PCA, and 2% MEA are shown in Fig. 1 and described under the respective species. The different shapes of conidia with aseptate, 1-septate, two-septate and muriform shapes confirmed that isolate “PTD-MA” belongs to *N. novaehollandiae* (Fig. 2) based on the key prepared according to previous studies^1,34,35^:

- Conidia formed within a pycnidium (Coelomycetous synasexual morph present).
- Conidia formed as dry powdery arthric chains (Coelomycetous synasexual morph absent): *Neoscytalydium* genus.
- Conidiomata semi-immersed or superficial with muriform conidia: *Neoscytalidium novaehollandiae.*
- Conidiomata immersed, eventually erumpent, conidia central cell dark brown, end cells hyaline to pale brown: *Neoscytalidium dimidiatum.*
- Conidia hyaline (conidia of coelomycetous synasexual morph hyaline): *Neoscytalidium orchidacearum.*
- Conidia occurring only in arthric chains in aerial mycelium: *Neoscytalidium oculus.*

Despite similarity among four *Neoscytalidium* species, the diagnostic morphological characters of *N. novaehollandiae* are having both two-septate (Fig. 2j–n) and muriform (Fig. 2o,p) conidia, while *N. dimidiatum*, *N. orchidacearum* and *N. oculus* lack these characters. So, morphological investigations showed that isolated fungi belonged to *N. novaehollandiae.*

### Phylogenetic analysis

**Figure 3:**
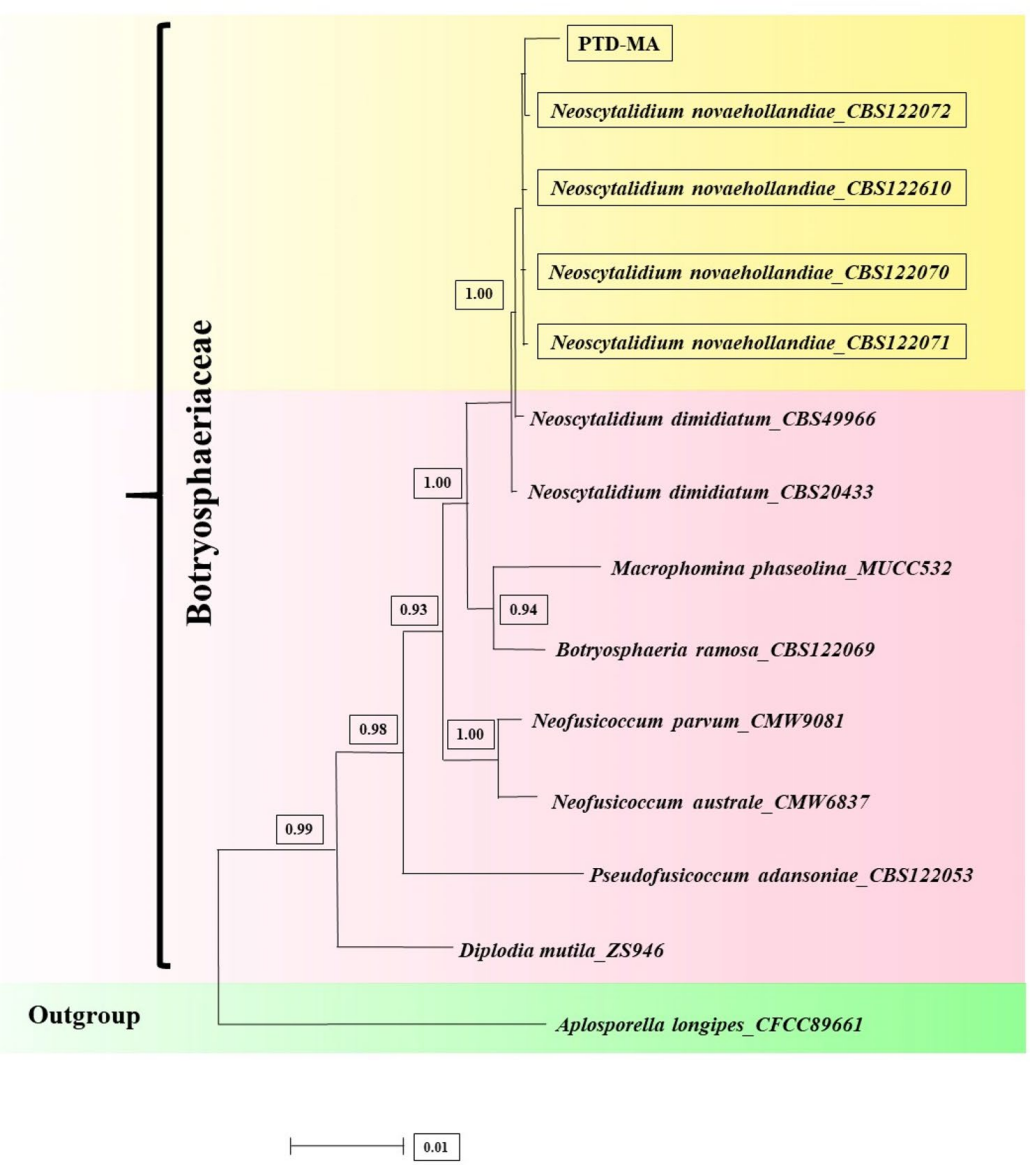
Bayesian tree inferred under the GTR + G model from the combined sequences of ITS and TEF1-α for *Neoscytalidium novaehollandiae* and related species of closest genus, using *Aplosporella longipes*^71^ as outgroup. The sequences aligned using MAFFT software and edited using Gblocks program. Bayesian posterior probabilities more than 0.50 are given for appropriate clades. Newly obtained sequence and other isolates of *N. novaehollandiae* indicated by yellow framework. PTD-MA is the strain of this study.

**Figure 4:**
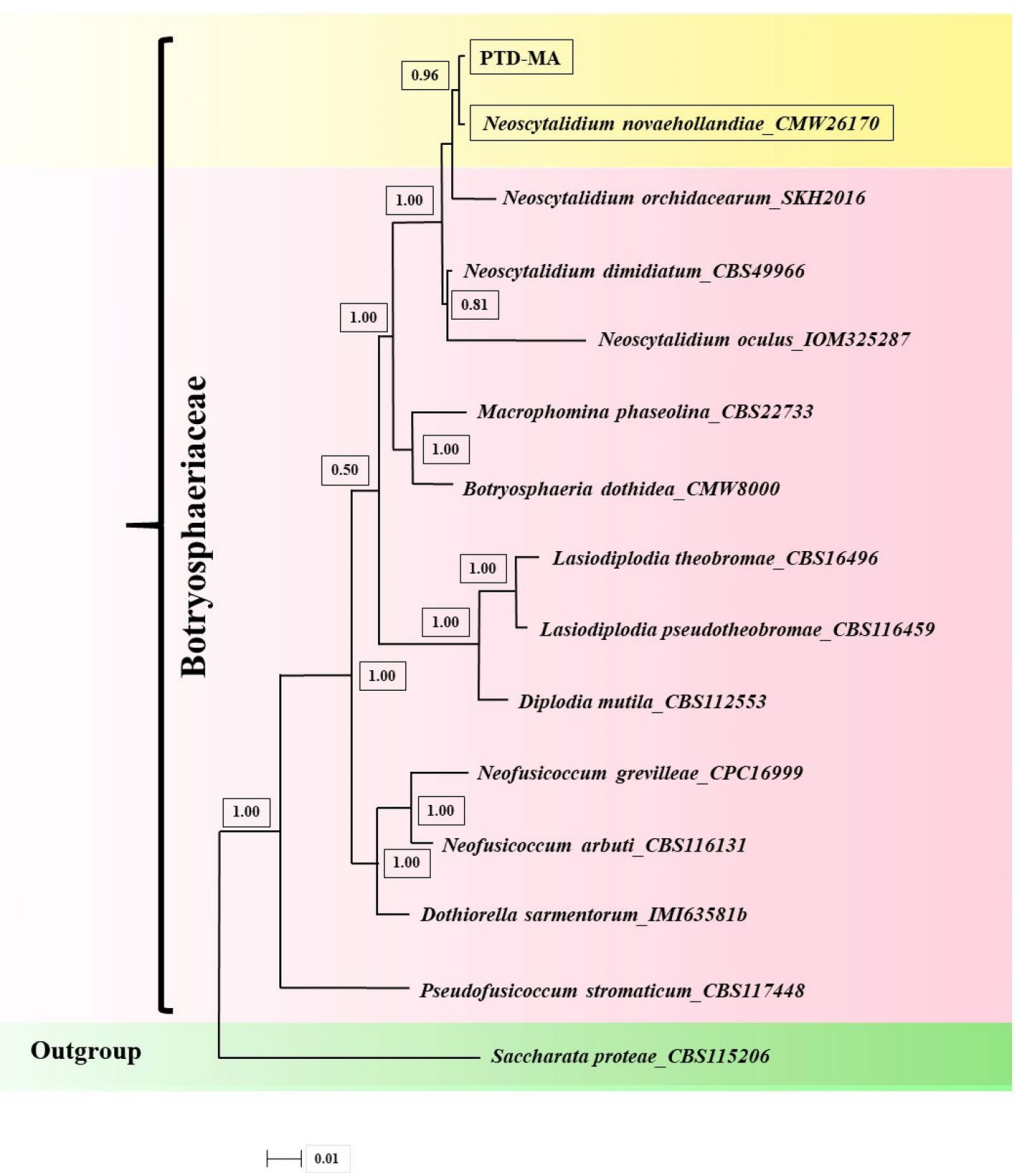
Bayesian tree inferred under the SYM + G model from the combined sequences of ITS and LSU for *Neoscytalidium novaehollandiae* and related species of closest genus, using *Saccharata proteae* as outgroup. The sequences aligned using MAFFT software and edited using Gblocks program. Bayesian posterior probabilities more than 0.50 are given for appropriate clades. Newly obtained sequence and another isolate of *N. novaehollandiae* indicated by yellow framework. PTD-MA is the strain of this study.

**Figure 5:**
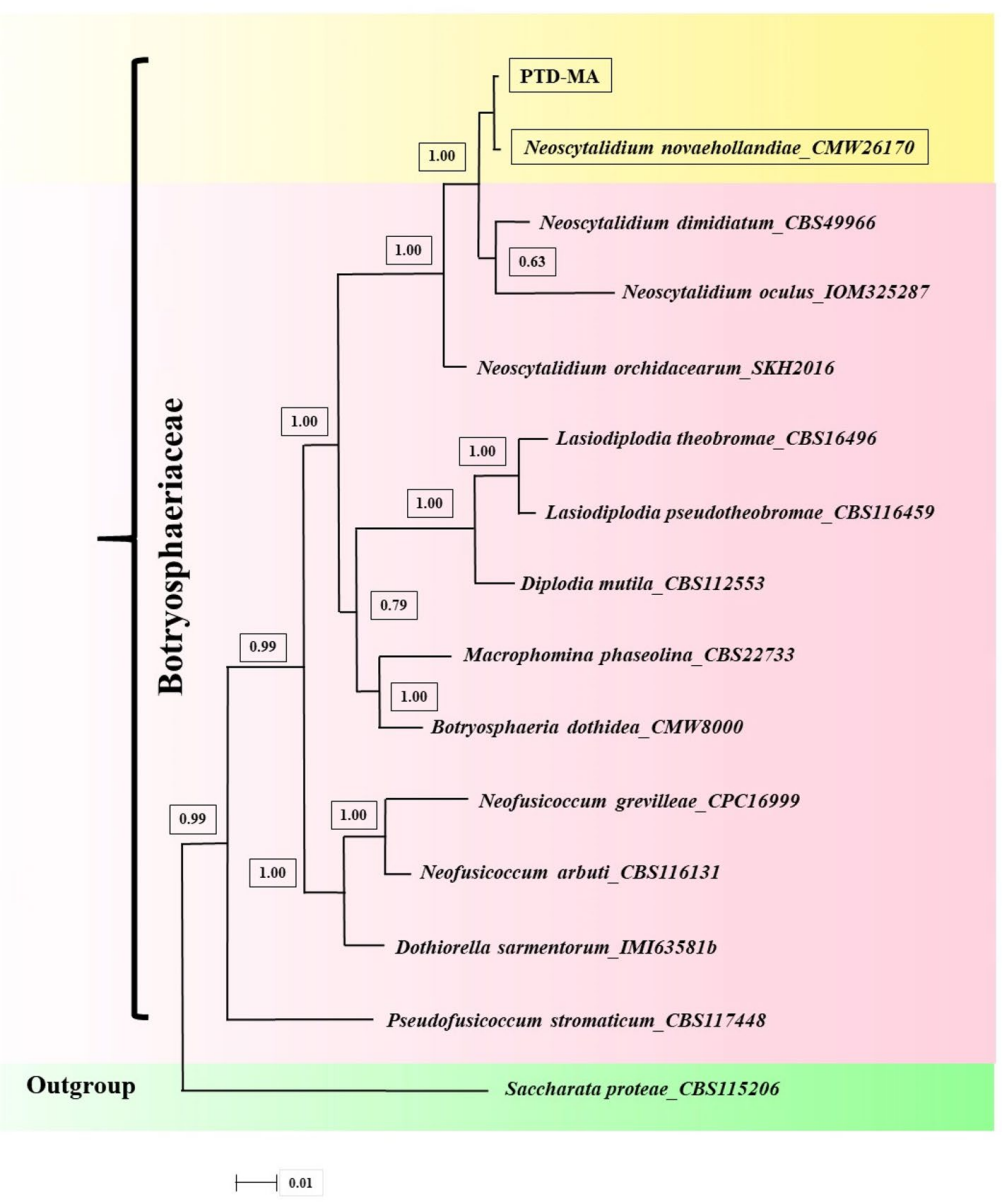
Bayesian tree inferred under the GTR + G model from the combined sequences of ITS, TEF1-α and LSU for *Neoscytalidium novaehollandiae* and related species of closest genus, using *Saccharata proteae* as outgroup. The sequences aligned using MAFFT software and edited using Gblocks program. Bayesian posterior probabilities more than 0.50 are given for appropriate clades. Newly obtained sequence and another isolate of *N. novaehollandiae* indicated by yellow framework. PTD-MA is the isolate of this study.

To specify the phylogenetic affinities of strain “PTD-MA” and the closest species and genus (Table 2), two separate phylogenetic trees with combined sequences of ITS_TEF-1α (dataset1), ITS_LSU (dataset2) and ITS_TEF-1α_LSU (combined sequences of dataset1 and 2) were organized for Bayesian inference (BI). For ITS_TEF-1α phylogeny (Bayesian tree), a total number of 28 sequences were selected including the newly generated sequences and sequences of *N. novaehollandiae, N. dimidiatum, Neofusicoccum parvum, N. austral, Macrophomina phaseolina, Pseudofusicoccum adansoniae, Diplodia mutila, Botryosphaeria_ramosa,* and *Aplosporella longipes* (as outgroup). Additionally, for ITS_LSU phylogeny (Bayesian tree), a total number of 30 sequences were selected including the newly generated sequences and sequences of *N. novaehollandiae, N. dimidiatum, N. oculus, N. orchidacearum, Neofusicoccum grevilleae, N. arbuti, Macrophomina phaseolina, Lasiodiplodia pseudotheobromae, Dothiorella sarmentorum, Diplodia mutila, Botryosphaeria dothidea, Pseudofusicoccum stromaticum, and Saccharata proteae* (as out group). Comparisons of combined sequences of ITS_TEF-1α (Fig. 3), combined sequences of ITS_LSU (Fig. 4) and combined sequences of ITS_TEF-1α_LSU (Fig. 5) of isolate “PTD-MA” with sequences in GenBank affirmed that the present species in this study belongs to *Neoscytalidium novaehollandiae*. ITS, TEF-1α and LSU sequences of isolate “PTD-MA” were deposited at NCBI under accession numbers MW605153, MW605154, and MW605155, respectively.

### Pathogenicity tests

**Figure 6:**
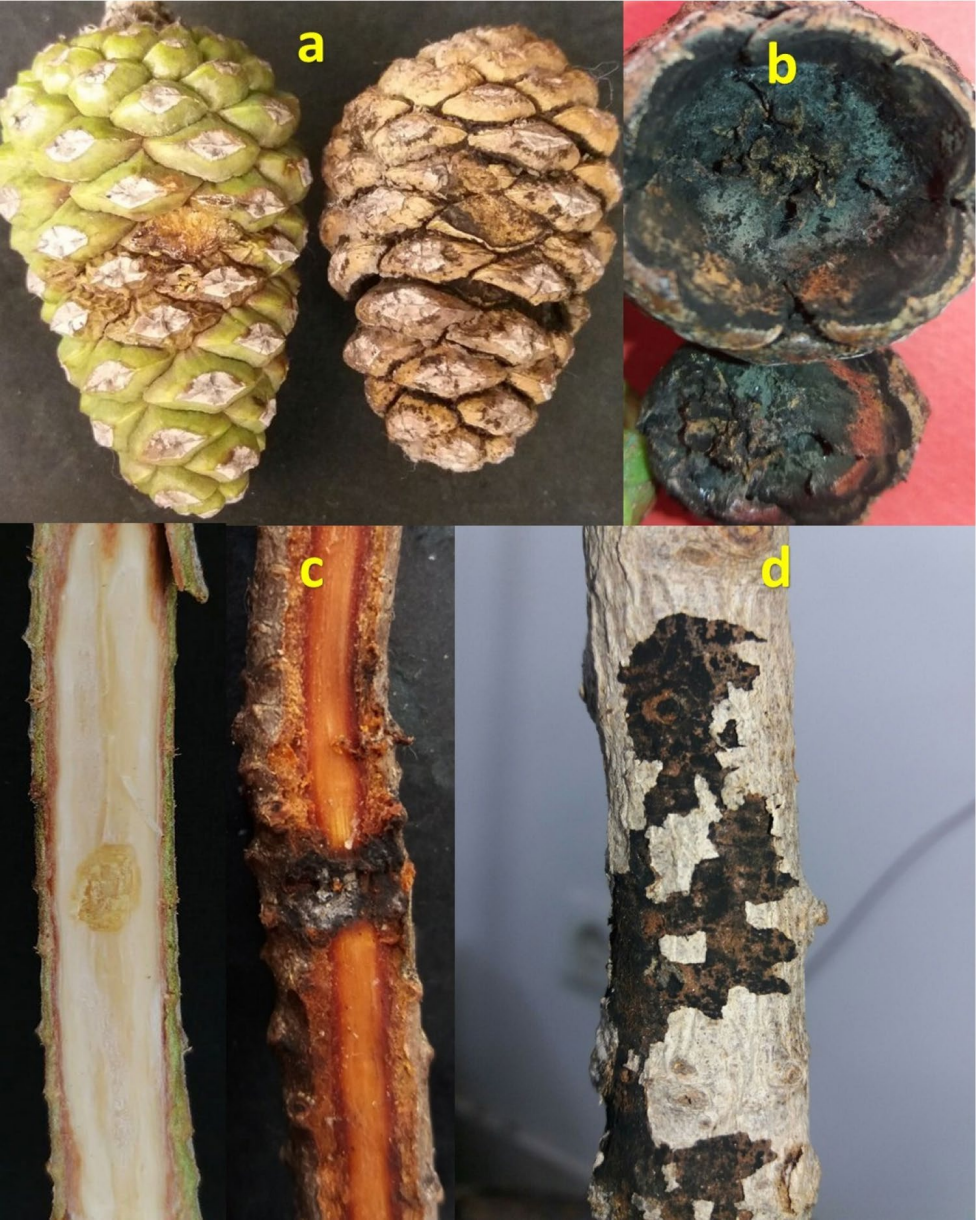
Pathogenicity tests. Negative control after inoculation with PDA (**a**-left); symptom on the fruit of pine after inoculation with the fungal disc (**a**-right); black spores of *Neoscytalidium novaehollandiae* with fruit separation of each other after 20 days (**b**); negative control (**c**-left); symptom on detached branches after 40 days (**c**-right); five-year-old trees treated with *N. dimiditum* black spores after 60 days (**d**). After annihilating the host tree, the fungi produced an asexual stage and tore the bark to spread.

Forty days post inoculation, the pathogenicity tests on the detached branches and fruits in pine showed the brownish chlorotic lesions and chlorotic tissues around inoculation point with black spores, respectively (Fig. 6a–c). The average lesion length was 19 ± 1 cm one month after inoculation, while no disease symptoms were observed in controls. Two months after artificial inoculation on pine saplings, the tree dried, and then the tree bark was torn, and black spores were appeared on the bark (Fig. 6d). All of the controls displayed no symptoms. The causal agent was successfully recovered, approving Koch’s postulates. The high mass of black spores produced. It is concluded that they can be air-borne and infect the same hosts or non-host plants in natural conditions.

### Quantitative analyses of disease

**Figure 7:**
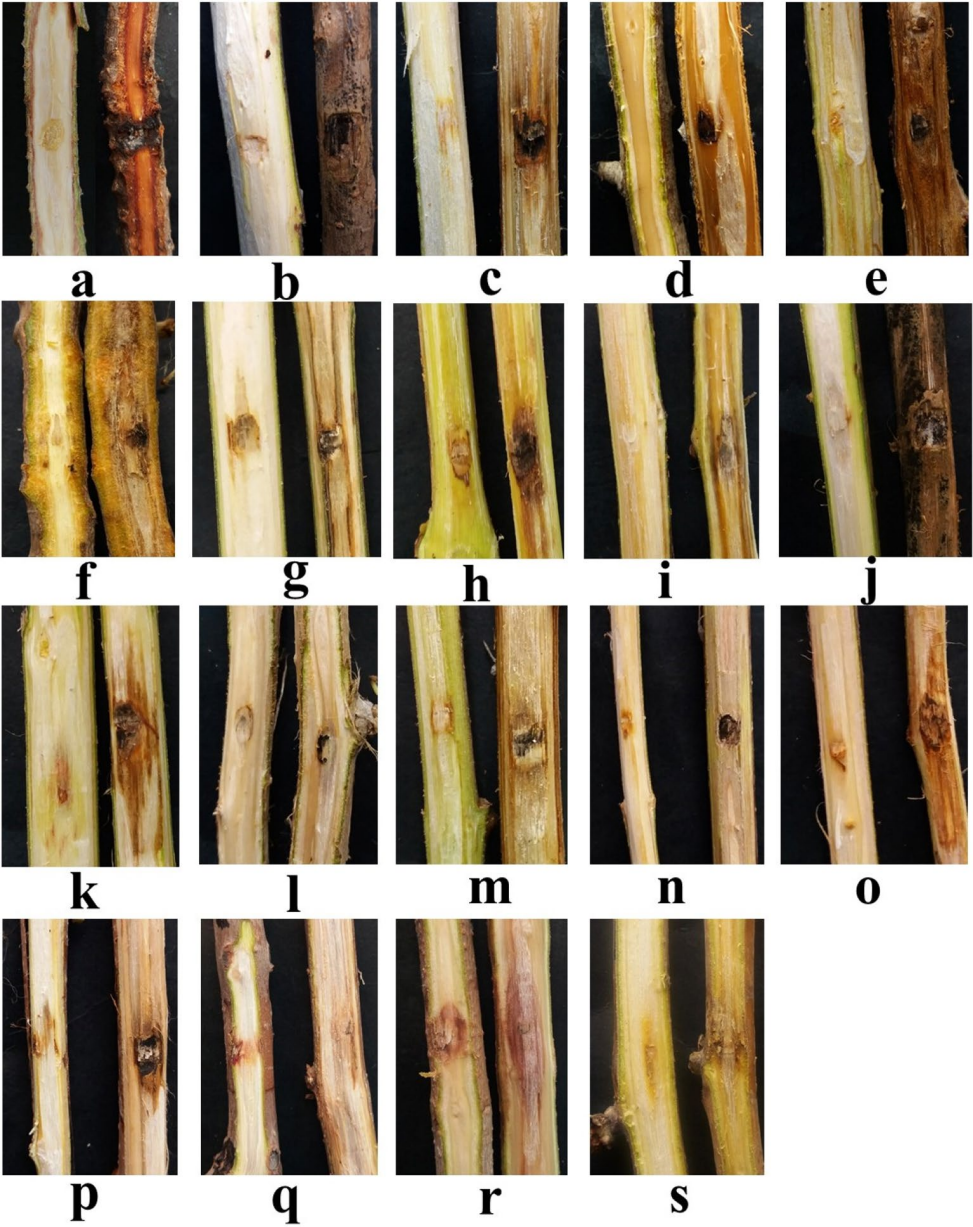
Pathogenicity tests on two-year old branches. There are two parts in each picture; negative control without symptom (left) and detached branch with lesion created by pathogen (right). Pine (**a**); willow (**b**); walnut (**c**); cypress (**d**); apricot (**e**); magnolia (**f**); ash (**g**); grape (**h**); chinaberry (**i**); poplar (**j**); pomegranate (**k**); ginkgo (**l**); sycamore (**m**); maple (**n**), elm (**o**); mulberry (**p**), catalpas (**q**), olive (**r**); ailanthus (**s**). All images are related to the lesions at 19–29 °C.

The results of three temperature and RH regimes (A, B, and C) showed the significant effects of high temperature and low humidity on the detached branches in 19 hosts. In C regime, the pathogen affected all plant species, and the inoculated tissues changed to brownish lesions (Fig. 7). The highest percentage of disease severity (PDS) discerningly belonged to willow, fig, apricot and mulberry with 100%. In B regime, 12 hosts displayed the symptoms which highest PDS belonged to apricot with 98%. Seven hosts including ash, grape, chinaberry, poplar, maple, elm and mulberry showed no symptoms, resistant to this pathogen in this regime. Furthermore, in A regime, 13 hosts showed symptom which highest PDS belonged to apricot with 89%. Additionally, 5 hosts including ash, grape, chinaberry, poplar, and elm showed no symptoms and considered as resistant hosts to this pathogen in this regime. Based on our knowledge, the results proposed that the high temperature and low humidity can play an essential role in host range of this fungal pathogen, so that temperature increment and relative humidity decrement contribute to establish it on other hosts.

**Figure 8:**
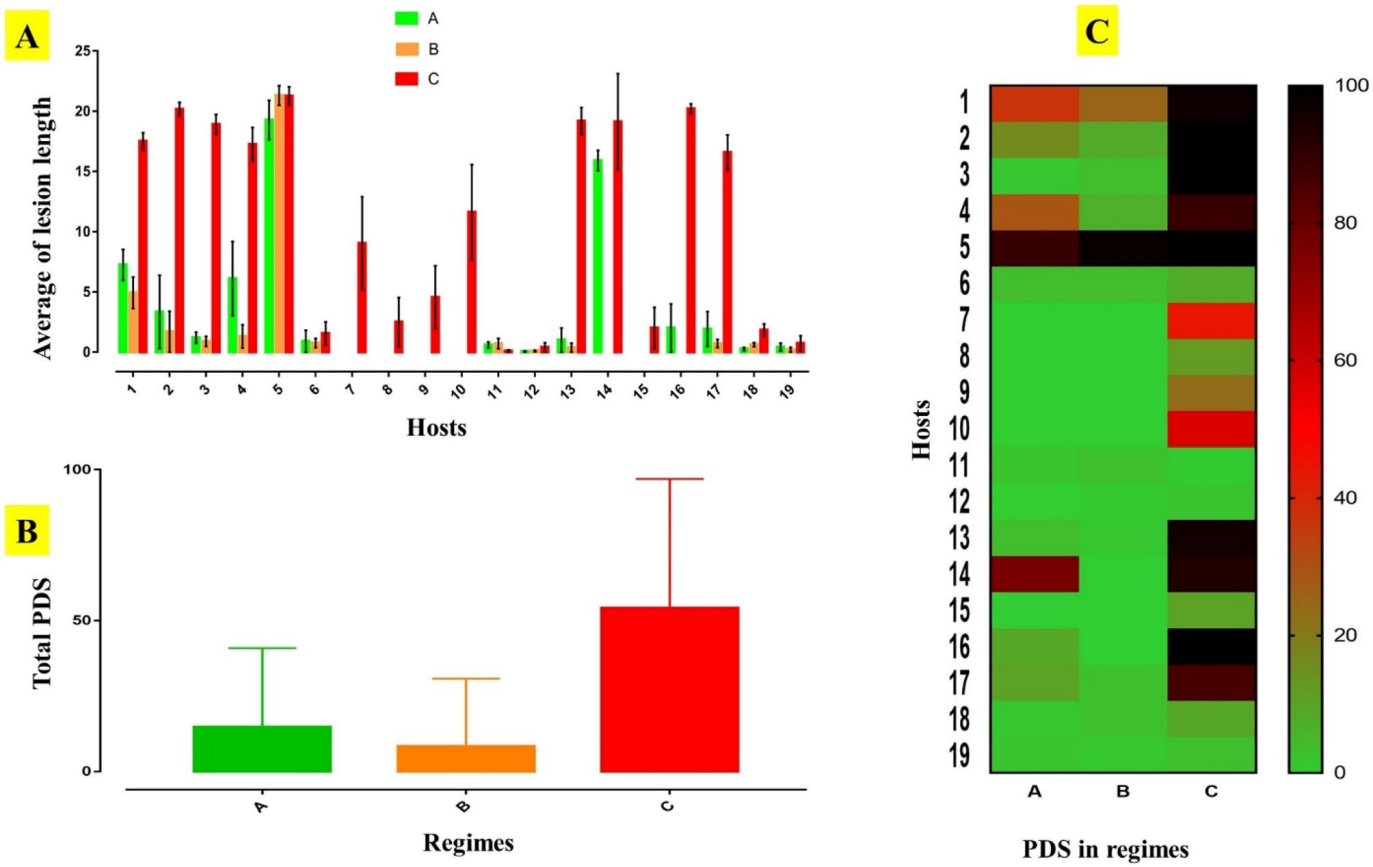
(**A**) Average of lesion length in all hosts in three regimes in regard to temperature and relative humidity (RH) “A (19 °C and 35% RH), B (24 °C and 80% RH) and C (29 °C and 15% RH. (**B**) Total PDS in three different regimes. (**C**) Heatmap of all hosts with their PDS in three regimes. The numbers belonged to hosts: 1-Pine; 2-willow; 3-fig; 4-cypress; 5-apricot; 6-magnolia; 7-ash; 8-grape; 9-chinaberry; 10-poplar; 11-pomegranate; 12-ginkgo; 13-sycamore; 14-maple; 15-elm; 16-mulberry; 17-catalpas; 18-olive and 19-ailanthus.

Based on the total average of lesion lengths in three conditions, the highest amounts belonged to C (10.75), A (3.11), B (1.74) regimes, respectively. Additionally, the total percentage of disease severity were recorded for C (15.63), A (8.87), and B (54.17) regimes, respectively. The highest PDS on detached branches of 19 hosts was recorded in C regime (high temperature and low humidity) (Fig. 8A). Total PDS were calculated for three regimes which C regime had the highest mean (Fig. 8B). The heatmap of 19 hosts displayed more PDS of *N. novaehollandiae* in C regime than A and B ones (Fig. 8C). Total scales related to all host responses to three regimes showed the susceptible and resistant condition in hosts affected by pathogen (Fig. 8A). These graphs clearly verified that high temperature and low humidity led to more PDS i.e., more damage to inoculated detached branches.

## Discussion

This study was performed for the first time to identify the dieback causal agent in pine trees in Tehran and Qazvin provinces. Causal agent of pine was identified as *N. novaehollandiae* according to morphological and molecular (ITS, TEF-1α and LSU sequences) studies^1,34,35,39,71^*.* The interesting point of this pathogen is that it may survive in spilled fruits at the foot of trees. Since this pathogen produces high populations of black powdery spores under torn skin, it can be air-born and infect other susceptible, injured or weak hosts.

*N. novaehollandiae* was reported in four provinces of Iran including Sistan and Baluchestan, Kermanshah, Gilan and Kerman on mulberry (*Morus alba*), black hawthorn (*Crataegus pentagyna*), hornbeam (*Carpinus betulus*), beech (*Fagus orientalis*), and oak (*Quercus brantii*)^36–38^. Furthermore, the pathogenicity of this species was confirmed on plants in China, Turkey and Australia which infected baobab (*Adansonia gibbosa*), bardi bush (*Acacia synchronica*), blue grevillea (*Grevillia agrifolia*), rattlepod (*Crotalaria medicaginea*)^39^, mango (*Mangifera indica*)^40,41^, elm (*Ulmus densa*)^42^, Grapevine (*Vitis vinifera*)^43^, pistachio (*Pistacia vera*)^44^, almond (*Prunus dulcis*)^45^, japanese persimmon (*Diospyros kaki*)^46^, tomato (*Solanum lycopersicum*(^47^, pear (*Pyrus communis*)^48^ and sage (*Salvia officinalis*)^49^. Based on the results, the host range of *N. novaehollandiae was* confined to 12 species of the plant hosts and most of them were woody plants. This study represents the first report of this species on *P. eldarica* worldwide. The present study showed the pathogen potential for attacking pine trees in Tehran and Qazvin provinces. So, to date, this pathogen was reported in six provinces of Iran including Tehran, Qazvin, Kerman, Kermanshah, Gilan and Sistan-Baluchestan^36–38^.

The pathogenicity tests on the detached branches and five-year-old tree confirmed that this pathogen can make brown lesions and decline symptoms associated with black spores of fungi at 29 °C. In another study, the pathogenicity of *N. novaehollandiae* was confirmed on *Quercus brantii* detached branches (incubated on a 12/12 h light/dark cycle at 25 °C for 25 days) and greenhouse pathogenicity tests on 2-year-old seedlings (natural day/night length, 25–27 °C, 60–70% RH), while no symptoms were observed on the mock-inoculated controls including *Ficus carica*, *Acer monspessulanum*, *Crataegus aronia*, *Amygdalus scoparia*, *Cornus mas*, *Pistacia atlantica* and *Pinus eldarica*^37^. The pathogenicity tests on branches of selected forest trees (12–15 years old) showed that lesion lengths varied between species including *Punica granatum, Alnus glutinosa, Pterocarya fraxinifolia, Parrotia persica, Mespilus germanica* and *Quercus castaneifolia*^38^*.* Furthermore, other studies briefly described the pathogenicity of *N. novaehollandiae on Morus alba*, *Crataegus pentagyna*, *Carpinus betulus*, *Fagus orientalis*, *Quercus brantii*, *Adansonia gibbosa*, *Acacia synchronica*, *Grevillia agrifolia*, *Crotalaria medicaginea*, *Mangifera indica, Ulmus densa*, *Vitis vinifera*, *Pistacia vera*, *Prunus dulcis*, *Diospyros kaki*, *Solanum lycopersicum, Pyrus communis*, and *Salvia officinalis*^36–42,44–49^.

This study assessed the response of the detached branches of pine, willow, cypress, apricot, magnolia, fig, ash, grape, chinaberry, poplar, pomegranate, ginkgo, sycamore, maple, elm, mulberry, catalpas, olive, and ailanthus against *N. novaehollandiae* under three temperature and relative humidity regimes for the first time. The application of several growth chambers made it possible to set three temperature and relative humidity regimes to gain reliable results. Likewise, more investigations affirmed the susceptibility of various cultivars and hosts to pathogenicity assays in controlled conditions. Mideros et al.^50^ verified the pathogenicity of *Phytophthora betacei* on four cultivars of tamarillo (*Solanum betaceum*). Besides, 19 species of *Phytophthora* caused different lesion sizes on 15 on urban forest tree hosts including *Agonis flexuosa*, *Banksia sessilis*, *Callistemon* sp., *Corymbia calophylla*, *Eucalyptus gomphocephala*, *E. marginata*, *Ficus microcarpa*, *Fraxinus excelsior*, *Magnolia grandiflora*, *Melaleuca* sp, *Metrosideros excels*, *Olea europaea*, *Platanus orientalis*, *Pyrus ussuriensis*, and *Viburnum tinus*^51^. *Lasiodiplodia theobromae*, as decline pathogen, was evaluated to diagnose the susceptible varieties in mango trees, and the results revealed that most of the mango varieties were susceptible to this pathogen except Bagan Pali, Saroli and Saleh Bhai^52^. Also, the cultivars of olive were exposed to *Verticillium dahlia* to determine the resistance or susceptible cultivars. Most cultivars exhibited the susceptibility except two genotypes (Kalamon and Koroneiki)^53^. Similar investigations have shown the pathogenicity test of *Diplodia bulgarica* on apple cultivars divided the cultivars into most susceptible and moderately resistant groups^54^. Although the optimum conditions for most fungal pathogens are the combination of high temperature and high humidity^55^. This research and other studies showed that *N. dimidiatum*^56–60^ and even *N. novaehollandiae* can progressively grow and make symptoms in high temperatures and low relative humidity. An interesting point of this study is the determination of the susceptible hosts. So, the plantation of these susceptible hosts will be avoided in regions with high infections. Conclusively, this study presents the valuable information towards novel researches on both *P. eldarica* and *N. novaehollandiae*. More investigations are required to characterize factors promoting diseases caused by *N. novaehollandiae*. This can notify the strategies to prevent and manage diseases caused by this pathogen not only in *P. eldarica* but also in other woody plants and crops in the different regions of Iran.

## Material and methods

### Sampling and pathogen isolation

**Figure 9:**
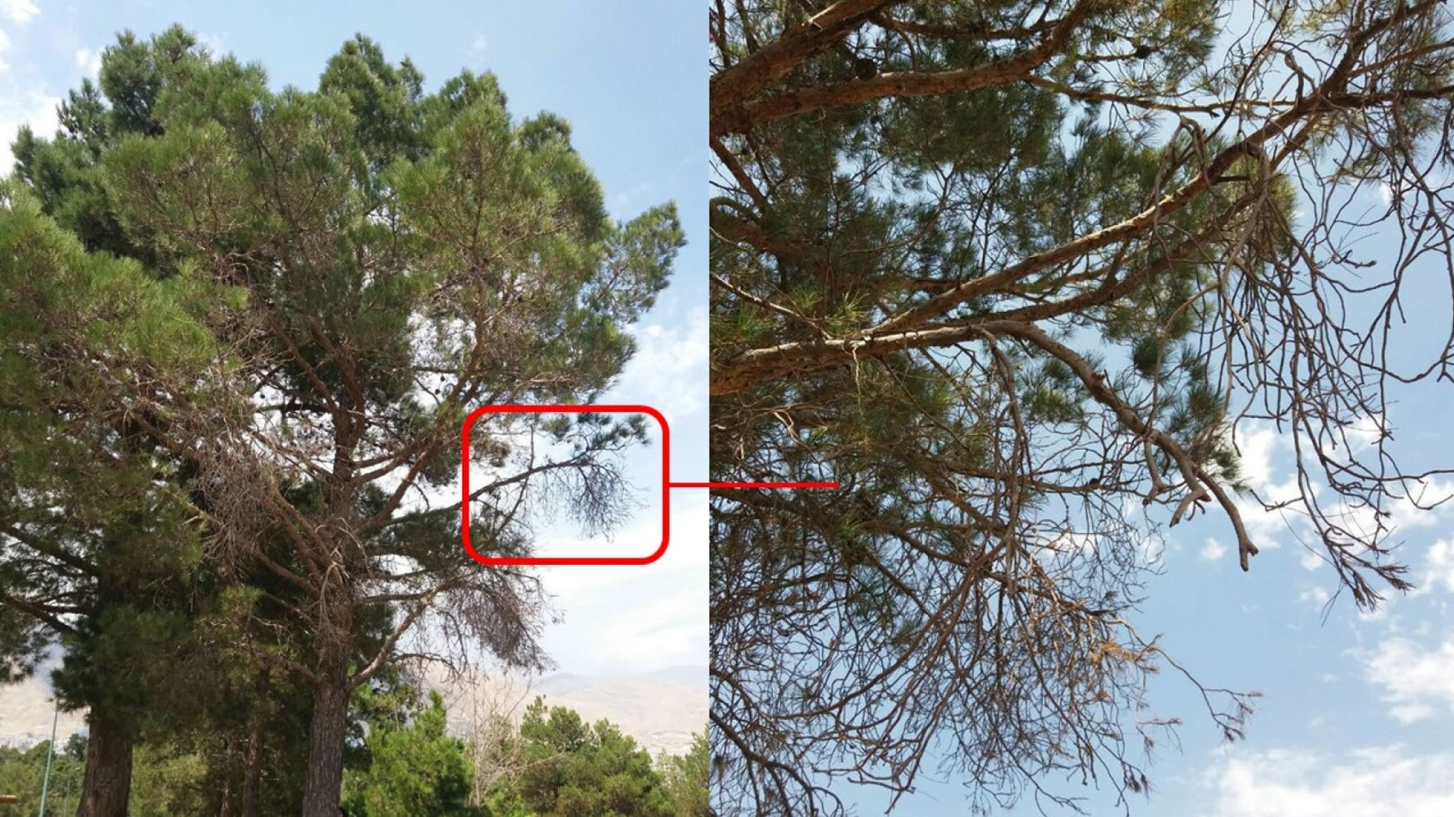
Dieback symptom on *Pinus eldarica* tree.

The symptoms of the decline and dieback were observed on the pine trees in Tehran (35°43′48″ N, 51°12′35″ E and 35°44′16″ N, 51°09′52″ E) and Qazvin (36°18′50″ N, 49°58′26″ E and 36°19′15″ N, 49°58′31″ E) provinces in 2019 (Fig. 9). The symptomatic fruits and branches of the pines were sampled in paper envelopes and transferred to the laboratory and kept at 5 °C. The infected tissue was cut into 1 × 2 cm pieces by sterilized gardening scissors, dipped in 70% ethanol for 80 s, and washed two times with sterile distilled water for 1 min. These pieces were placed in sterilized blotting paper to eliminate excess water. Four to six pieces of surface-sterilized infected parts were cultured on Potato dextrose agar (PDA) plates supplemented with Tetracycline 20% and incubated in dark at 27 °C for seven days for the growth of the fungi out of infected tissues. The grown mycelia were transferred to a fresh PDA plate. New subcultures were purified by hyphal tip culture and used for further investigations. The isolate was deposited in the plant pathology department at Tarbiat Modares University.

### Plant materials

It is notified that 10–20-year-old pine trees were legally samples from unprotected and forestry regions with municipal support and recommendation, and all methods comprising plant studies were performed in accordance with the relevant guidelines, regulations and legislations. Required permission for collecting pine plant materials was obtained.

### Morphological characterizations and media cultures

Fungal isolates were grown on 9 cm Petri dishes on standard media, i.e., potato carrot agar (PCA), oatmeal agar (OA), potato dextrose agar (PDA), and malt extract agar (MEA; 2% malt extract, 6 g peptone, and 15 g agar) for morphological studies. The fungal slide preparations were examined using an Olympus BX51 Microscope. Arthroconidia and different types of conidia of the anamorph were documented according to Pavlic et al. method^39^.

### DNA extraction, PCR amplification and sequencing

Genomic DNA was extracted from one representative isolate as described previously^61–63^. Briefly, the isolate was grown on potato dextrose broth (PDB) for 8 days at 27 °C and genomic DNA was extracted from the fungal colony. The fungal mycelia were ground under liquid nitrogen in a mortar and pestle. Then 40–100 mg of ground mycelia transferred to a 1.5 mL microtube and 400 μL of DNA salt solution (Tris–HCl, 100 Mm; EDTA, 5 Mm; NaCl, 1.4 M; pH, 7.5–8) was added and mixed thoroughly with a vortex. This microtube was transferred to a 65 °C water bath for 15 min, and then was incubated at ice for 15 min. Then the homogenate was centrifuged for 10 min at 10,000 rpm at 4 °C. The supernatant was transferred to a new tube and then cold isopropanol (0.7 its volume) was added to it. The tube was slowly vortexed several times for 4 s. The tube was centrifuged at 20 °C for 15 min at 10,000 rpm. The supernatant was slowly decanted without disturbing the pellet and placed at 25–30 °C for 40–60 min to remove alcohol. The pellet was dissolved in 30 µl deionized water.

Internal transcribed spacer (ITS) region, large*-*subunit (LSU) region and translation elongation factor 1-α (TEF1-α) region were amplified using ITS1/ITS4^64^, LROR/LR7^65^ and EF1-728F/EF1-986R^66^ primers, respectively (Table 1). PCR reaction (25 µl) included 12.5 µl Mastermix, 9 µl deionized water, 1 µl Forward primer, 1 µl Reverse primer, and 1.5 µl DNA. The PCR procedure for ITS and LSU was as follows: initial denaturation at 95 °C for 3 min, followed by 35 cycles at 94 °C for 40 s, 58 °C for 45 s, and 72 °C for 1 min, and a final extension of 72 °C for 10 min. Furthermore, the PCR procedure for EF1-α was as follows: initial denaturation at 96 °C for 3 min, followed by 35 cycles at 95 °C for 30 s, 54 °C for 45 s, and 72 °C for 45 s, and a final extension of 72 °C for 7 min. The PCR product was analyzed on a 1% agarose gel stained with GreenView Ultra. The PCR products were purified and sequenced by Biomagic Company (Karaj-Iran).

**Table 1:** Sequences of the primers used for PCR analysis.

	Regions	Primer names	Primer sequences	References
1	Internal transcribed spacer (ITS)	ITS1	5′-TCCGTAGGTGAACCTGCGG-3′	^64^
		ITS4	5′-TCCTCCGCTTATTGATATGC-3′	
2	*Large-subunit* (LSU)	LROR	5′-ACCCGCTGAACTTAA GC-3′	^65^
		LR7	5'-TAC TAC CAC CAA GAT CT-3′	
3	Translation elongation factor 1-α (TEF 1-α)	EF1-728F	5′-CATCGAGAAGTTCGAGAAGG-3′	^66^
		EF1-986R	5′-TACTTGAAGGAACCCTTACC-3′	

### Phylogenetic analysis

The newly sequenced regions were observed with chromas software (www.technelysium.com.au/chromas.html) for picks of nucleotides and then were manually edited. The Basic Local Alignment Search Tool (BLAST) (https://blast.ncbi. nlm.nih.gov/Blast.cgi) was employed to compare the new ITS, TEF-1α, and LSU sequences with other sequences accessible in GenBank database. The datasets of combined sequences of ITS_TEF-1α and ITS_LSU sequences were separately aligned using the Q-INS-i algorithm of an online version of MAFFT v.7.205 (https://mafft.cbrc.jp/alignment/server/)^67^. The Gblocks program (version 0.91b) with all the three less stringent parameters (Allow smaller final blocks, Allow gap positions within the final blocks, and Allow gap positions within the final blocks) a server tool at the Castresana Lab (http://molevol.cmima.csic.es/castresana/ Gblocks_server.html) was utilized for editing of the alignments, i.e., to remove the regions with poor align or divergent positions. The combined sequences of ITS_TEF-1α and ITS_LSU aligned and edited in this manner were called dataset1 and dataset2, respectively. The best-fitting models for dataset1 and dataset2 were chosen using PAUP*/MrModeltest.2^68^. Bayesian analyses were carried out using MrBayes 3.1.2^69^ with a starting random tree and GTR + G model for ITS_TEF-1α (dataset1) and SYM + G model for ITS_LSU (dataset2) with four million generations. Dendroscope V.3.2.8 was used for the visualization of output files prepared by phylogenetic programs^70^. Dataset1 and dataset2 are indicated at Table 2.

**Table 2:** Fungal isolates and GenBank accession numbers of taxa used in the phylogenetic analyses.

Dataset1				Dataset2			
Strain	Species	ITS	TEF1-α	Strain	Species	ITS	LSU
PTD-MM	*Neoscytalidium novaehollandiae*	MW605153	MW605154	PTD-MM	*N. novaehollandiae*	MW605153	MW605155
CBS122072	*Neoscytalidium novaehollandiae*	EF585535	EF585581	IOM 25,287	*N. oculus*	MG764431	MG764432
CBS122610	*Neoscytalidium novaehollandiae*	EF585536	EF585578	MFLUCC 12-0533e	*N. orchidacearum*	KU179865	KU179864
CBS122070	*N. novaehollandiae*	EF585539	EF585579	CMW 26,170	*N. novaehollandiae*	KF766207	KF766374
CBS122071	*Neoscytalidium novaehollandiae*	EF585540	EF585580	CBS 499.66	*N. dimidiatum*	KF531820	DQ377925
CBS499.66	*Neoscytalidium dimidiatum*	AY819727	EU144063	CBS 129,518 ex-type	*Neofusicoccum grevilleae*	JF951137	JF951157
CBS204.33	*Neoscytalidium dimidiatum*	AY819728	EU144064	CBS 227.33	*Macrophomina phaseolina*	KF531825	DQ377906
CMW9081	*Neofusicoccum parvum*	AY236943	AY236888	CBS 164.96 ex-neotype	*Lasiodiplodia theobromae*	AY640255	EU673253
CMW6837	*Neofusicoccum australe*	AY339262	AY339270	CBS 116,459 ex-type	*Lasiodiplodia pseudotheobromae*	EF622077	EU673256
MUCC532	*Macrophomina phaseolina*	EF585505	EF585560	CBS 116,131 ex-type	*Neofusicoccum arbuti*	AY819720	DQ377915
CBS122053	*Pseudofusicoccum adansoniae*	EF585525	EF585569	IMI 63581b ex-type	*Dothiorella sarmentorum*	AY573212	AY928052
ZS94-6	*Diplodia mutila*	AF243407	AY236904	CBS 112,553	*Diplodia mutila*	AY259093	AY928049
CBS122069	*Botryosphaeria_ramosa*	EU144055	EU144070	CBS 115,476 ex-epitype	*Botryosphaeria dothidea*	AY236949	AY928047
CFCC89661	*Aplosporella longipes*	KM030583	KM030597	CBS 117,448 ex-type	*Pseudofusicoccum stromaticum*	AY693974	DQ377931
				CBS 115,206	*Saccharata proteae*	KF766226	DQ377882

Newly generated sequences (ITS, EF1-a, and LSU) in this study are specified in bold italics. These strains and accession numbers were obtained based on the several studies^1,34,35,39,71^.

### Pathogenicity tests

Inoculating detached branches is a standard test described by Afek et al.^72^ and researchers^73–76^. To clarify terminology, these was cited as in vitro, in vivo or *in planta* tests. To gain consistent test material, the detached branches (2 cm diameter) from healthy 2-year-old shade trees (pine, willow, walnut, cypress, apricot, magnolia, ash, grape, chinaberry, poplar, pomegranate, ginkgo, sycamore, maple, elm, mulberry, fig, catalpas, olive, and ailanthus) were selected and cut into 19–20 cm long pieces. A total of 342 fresh and healthy branches (285 pieces for pathogenicity tests and 57 pieces as negative control) were used for the pathogenicity tests^72^. Fungal isolates were grown on PDA medium for 8 days, and then the skin and a little part of the wood of the detached branches were removed and inoculated with a fungal disc (5 mm diameter). The control was inoculated with PDA. The lesions inoculated with fungal disc and PDA were sealed with Parafilm, and both ends were also sealed with wet cotton to prevent the desiccation of the detached branches and maintained in the growth chamber at 19 °C with 35% relative humidity (RH) for 20 days. After 20 days, these detached branches were divided into three parts, and they were placed in three different conditions in regard to temperature and RH, designated as A, B and C regimes, in the growth chamber for 20 days. These regimes were as follow: A regime (19 °C and 35% RH), B regime (24 °C and 80% RH) and C regime (29 °C and 15% RH). The cotton pieces were wetted every other day to prevent the desiccation of the detached branches. Furthermore, a pathogenicity test was performed on five-year-old pine trees. Artificial lesions (5 cm long and 2 cm wide) were made on the skin and a part of wood in tree and inoculated as described above. Koch’s postulates were done to confirm fungal pathogenicity.

### Disease measurement

Lesion length was measured in all hosts based on color change to brownish necrosis on the detached branches. For the evaluation of the percentage of disease severity (PDS), lesion length was divided by branch length (Table 3.).

**Table 3:** The results of three regimes (A, B, and C) were the significant effects of high temperature and low humidity for detached branches in 19 hosts. In C regime, the pathogen affected all samples. It changed the inoculated tissues to brownish lesions.

Trait	Average lesion length in detached branches (cm)			Percentage of disease severity (PDS)		
Hosts	A	B	C	A	B	C
1-Pine	7.24	4.93	17.50	37	25	97
2-Willow	3.34	1.70	20.16	16	8	100
3-Fig	1.20	0.88	18.90	1	4	100
4-Cypress	6.10	1.30	17.25	29	7	89
5-Apricot	19.26	21.30	21.26	89	98	100
6-Magnolia	0.91	0.76	1.56	4	4	8
7-Ash	0.00	0.00	9.03	0	0	44
8-Grape	0.00	0.00	2.50	0	0	12
9-Chinaberry	0.00	0.00	4.56	0	0	23
10-Poplar	0.00	0.00	11.62	0	0	57
11-Pomegranate	0.60	0.70	0.10	2	3	0.4
12-Ginkgo	0.05	0.10	0.41	0	0	2
13-Sycamore	1.00	0.37	19.17	4	1	96
14-Maple	15.9	0.00	19.12	76	0	93
15-Elm	0.00	0.00	2.01	0	0	10
16-Mulberry	2.00	0.00	20.2	9	0	100
17-Catalpas	1.91	0.72	16.58	10	3	86
18-Olive	0.26	0.62	1.83	1	3	9
19-Ailanthus	0.42	0.20	0.74	2	1	3
Total average	3.11	1.74	10.75	15.63	8.87	54.17

### Statistical analysis

The experiment, containing all controls and different treatments, was performed in quintuplicate. The hypothesis of normality and equal variance were tested. Conventional parametric statistics applied for the analysis. The data was statistically analyzed by using SAS (SAS 9.1) and SPSS (SPSS 15.0). ANOVA was conducted by GLM statistical method and means comparison was done by least significant difference (LSD) test. GraphPad Prism (GraphPad Prism 5) software was used for making graphs.
